# Self-Supervised Sidewalk Perception Using Fast Video Semantic Segmentation for Robotic Wheelchairs in Smart Mobility

**DOI:** 10.3390/s22145241

**Published:** 2022-07-13

**Authors:** Vishnu Pradeep, Redouane Khemmar, Louis Lecrosnier, Yann Duchemin, Romain Rossi, Benoit Decoux

**Affiliations:** Normandie University, UNIROUEN, ESIGELEC, IRSEEM, 76000 Rouen, France; vishnu.pradeep@esigelec.fr (V.P.); louis.lecrosnier@esigelec.fr (L.L.); yann.duchemin@esigelec.fr (Y.D.); romain.rossi@esigelec.fr (R.R.); benoit.decoux@esigelec.fr (B.D.)

**Keywords:** video semantic segmentation, sidewalk segmentation, cross-domain, spatial convolution, dilated convolution, error mitigation, environment perception

## Abstract

The real-time segmentation of sidewalk environments is critical to achieving autonomous navigation for robotic wheelchairs in urban territories. A robust and real-time video semantic segmentation offers an apt solution for advanced visual perception in such complex domains. The key to this proposition is to have a method with lightweight flow estimations and reliable feature extractions. We address this by selecting an approach based on recent trends in video segmentation. Although these approaches demonstrate efficient and cost-effective segmentation performance in cross-domain implementations, they require additional procedures to put their striking characteristics into practical use. We use our method for developing a visual perception technique to perform in urban sidewalk environments for the robotic wheelchair. We generate a collection of synthetic scenes in a blending target distribution to train and validate our approach. Experimental results show that our method improves prediction accuracy on our benchmark with tolerable loss of speed and without additional overhead. Overall, our technique serves as a reference to transfer and develop perception algorithms for any cross-domain visual perception applications with less downtime.

## 1. Introduction

As scene perception has become a staple requirement within many computer vision applications, accurate and efficient object detection and classification is now on its core foundations. In recent years, the approaches using convolution neural networks (CNN) have produced impressive results in numerous perception problems including object detection [1,2], classification [3,4,5], and scene segmentation [6,7,8]. These approaches have also been used for end-to-end learning of robotic tasks such as detection of goals [9] and autonomous navigation [10]. In addition to standard benchmark metrics and the new end-to-end learning algorithms, they are yet to become the dependable solution for outdoor robotic perception. Generally, perception tasks in outdoor environments are coherently onerous due to frequently altering environments accompanied by the change in appearance throughout the day and across the seasons. Combining that with the void due to the unavailability of datasets encompassing these adversities to effectively train and validate the deep learning (DL)-based machine learning (ML) models leads to an improbable solution to the outdoor robot perception problem.

Theoretically, an approach to outdoor perception can be termed as generic if the dataset on which the models are evaluated are rich in diversity that ensure generalization to varying objects and scenarios and environmental conditions [11] and the same model is able to yield stable, reliable predictions over time. Usually the CNN layers are trained on such a data distribution by minimising a given loss function on the training set. Consequently they will perform well if the evaluation domain is similar to the training domain and is carried out on a per frame basis. However the inputs are normally image sequences from passive optical sensors while in use in real world applications, and therefore these mechanisms are prone to adventitious behaviours. Hence it is uniformly important to obtain a high level of accuracy in prediction and maintain a consistent behaviour while performing in heterogeneous environments.

As a consequence, a number of authors have proposed methods that can allow domain adaption (for example [11,12,13,14,15,16]) in cases where fully or partially labelled set of samples from fixed target distributions are available. Although these adapted algorithms can succeed in training models well on known target distributions, the presupposition of a prior fixed target distributions will possibly be a constraint in practical scenarios. For instance, consider a semantic segmentation algorithm deployed on a mobile robot. A new task, different camera arrangement or an unplanned environment, will alter the target distribution. A common issue as these diverse scenarios can only be identified after the model is trained and deployed [11].

Environment perception techniques for roads by using semantic segmentation is an acutely active area of innovation. In this paper, we aim to transfer these approaches to perform in a distinct domain, the sidewalk environment. Our experimental setup and results provide an insightful approach that could be used as a guideline for nearly all robotics projects that aim at developing a general robust visual perception technique for autonomous control, navigation, and maneuver in diverse environments.

Our robot is an electrically powered wheelchair (EPW), an auspicious, distinctive, challenging real-world use case equipped with two Intel RealSense cameras (the RGB-D D435 and tracking T265) and a NVIDIA Jetson TX1 single-board computer. The robot is a part of the ADAPT project that aims at extending the usability of EPWs among people with different degrees of impairment [17].

Inspired by the recent developments in video semantic segmentation (VSS) approaches, we propose a novel method for efficient video segmentation that include: (1) a base semantic segmentation model that adopts ResNet18 backbone [18,19]; (2) GSVNET [20], a propagation network that does guided spatially varying convolution on downscale crude feature maps from the base segmentation network; (3) a multi-scale dilated convolution to further enhance the receptive field of the feature map; and (4) a low-rank bilinear upscaling method to obtain the segmentation maps in the original resolution. To correspond with our distinctive use case we produce our own synthetic dataset with a blending target distribution to ablate, train, and validate our method while performing scene perception for our autonomous EPW.

The remainder of this paper is structured as follows. In Section 2, we review the related works in smart assist features for EPW and video semantic segmentation. In Section 3, we introduce our method that perform fast semantic segmentation (FSS). In Section 4, we present and explain our dataset, the benchmark metrics, and the analysis of the results we obtained. Finally, the conclusions and future directions are outlined in Section 5.

## 2. Related Work

Scene understanding techniques have an extensive scope to enable EPW users to commute more safely in challenging environments. In this section, we introduce related work in two fields namely, smart EPW assist features and lightweight video semantic segmentation.

### 2.1. EPW Smart Assist Features

With the emergence of embedded systems, significant innovations have occurred in EPWs that are triggered by the ambitions of several challenged people who use them. This includes users with multiple and/or severe disabilities who find it difficult to maneuver the wheelchair in typical places where approaches are tightly constrained [21] either due to various untypical and vivid outdoor settings or users’ lack of autonomy to control. Aside from these requirements on mobility, clinical surveys have also proved the users’ desire to gain leisure, independence, and productivity by benefiting from these technologies [22,23,24].

Intelligent wheelchair systems that can offer semi-autonomous to fully autonomous navigation systems based on computer vision utilizes a selection of goal techniques by using the head and gaze motion of users [25,26], eye movements [27,28,29], or a combination of speech and vision [30]. Moreover, technologies such as obstacle detection and collision avoidance can be used for better-assisted maneuvering of the wheelchairs. Zhengang et al. [31] proposed an ROS-based autonomous navigation approach by using an RGB-D camera for the environment and depth perception for obstacle avoidance, map building for unexplored areas by using an algorithm of target point generation based on edge detection. Viswanathan et al. [32] presented a real-time system that can detect and avoid obstacles by using stereo vision cameras and perform path planning using visual odometry.

Hengli et al. [33] addressed these issues in navigation for an EPW through tight spaces by proposing a self-supervised approach with a pipeline that automatically labels and captures frames for further fine-tuning their network. Seungbo et al. [34] used a CNN composed of only encoders to perform lightweight semantic segmentation to detect road surface damage detection. In [35], a residual network-based architecture called WideSeg [36] classifies the region of outdoor environments into sidewalks, crosswalks, and traffic lights.

### 2.2. Video Semantic Segmentation

Partitioning image segments into pixel-level regions belonging to various classes is called image semantic segmentation. The same process is carried on videos to obtain video semantic segmentation with various approaches [37,38,39] that would adjust the loss between speed and accuracy. Most of the methods apply the same CNN model to each frame and temporally aggregate the features with additional layers [40,41,42]. Although these methods score fine accuracy values over single frame approaches, they cause considerable overhead computation over a per-frame model.

Consequently, a few other approaches to leverage the overhead computational costs, proposed to maintain temporal continuity to propagate and reuse the high-level features from key frames [43,44,45,46]. However, the continuous motion of objects and their occlusion in videos are often challenging hindrances to robustly propagate pixel-level predictions over time. To tackle this problem, refs. [44,47] directly reuse relatively steady, high-level features extracted from downscaled frames in deep layers. Another approach is to suit optical flow to wrap high-level features in key frames to non key frames [43] and further update flow wrapped feature maps with shallow features extracted at current frames [46]. However, relying on optical flow causes significant computation costs and can fail with large variations and nontextured regions. To avoid using optical flow and reduce error propagation due to scene warping errors, Li et al. [45] uses a spatially varying convolution (SVC) together with a lightweight feature extractor for non-key frames.

Although these methods offer an overall computational costs reduction compared to their image segmentation baselines, evidently their accuracy have also decreased [43,45,46]. In addition, due to less reliable extractions at key frames, these methods are prone to inconsistent speeds with equivalent latency that of single-frame CNN models. Therefore, to perform fast, lightweight semantic segmentation on video, it is crucial to utilize the extracted features at the fullest potential and the feature extraction on non-key frames to be made sheer as possible.

GSVNET is a simple temporal propagation network that performs spatial convolutions on the segmentation maps obtained from a basic segmentation network [20]. The framework incorporates temporal wrapping based on optical flow on downscaled frames to estimate the feature maps of current frames, spatial convolutions to mitigate the errors from imperfect optical flows, and then concatenate the crude estimates from channels of a base segmentation network to form a downscaled segmentation of each frame. However, due to flow estimations on downscaled frames, the feature extraction suffers from a great degree of contextual information loss and therefore requires additional steps to mitigate this, while keeping the framework light as possible.

## 3. Fast Video Semantic Segmentation Method

### 3.1. GSVNET Semantic Segmentation Approach

For key frame segmentation, we use the SN-R18 and BN-R18 models, based on the SwiftNet-R18 [18] and BiSeNet [19] networks, and GSVNET as a propagation framework. GSVNET starts with a spatio-temporal estimation on downscaled segmentation obtained from the aforementioned models. $S_{t-1}$ is the segmentation from previous frame $I_{t-1}$. To arrive at an initial estimate $S_{t}$ for the current frame $I_{t}$, GSVNET performs temporal wrapping on $S_{t-1}$ based on optical flow, as expressed in Equation (1):

(1) $$S_{t}\left(c,x,y\right)=S_{t-1}\left(c,x+m_{tx},y+m_{ty}\right),\forall c\epsilon C$$

where *x* and *y* denote the pixel locations in the scaled-down segmentation and $m_{tx}$ and $m_{ty}$ are attributes from the optical flow network hierarchical feature fusion. To correct the errors during optical flow estimation, the segmentation wrapping is refined by applying a guided SVC. First, it performs a separable convolution on $S_{t}$ with several ideal kernel values to shift $S_{t}$ channel-wise by pixels in a certain direction and then sum it up with crude estimates from the current frame $I_{t}$. An SVC is applied across the channels of each chunk *C* and then summed up to obtain the downscaled estimate of the current frame’s segmentation $S_{t}\left(c,x,y\right)$.

### 3.2. Dilated Convolution

Dilated convolution helps in understanding the positional relationship and semantic relationship between objects [35]. We apply multi-scale convolution to the downscale segmentation map with 2,4,8 factors on $S_{t}\left(c,x,y\right)$ from GSVNET to refine the feature map. This can effectively extract global context information and also enlarge the receptive field without losing resolution [48,49,50]. Moreover, it uses sparse kernels to enlarge the receptive field without increasing the number of parameters or the computational costs.

The refined feature maps of different factors generated by dilated convolution are concatenated together with an input image. Through concatenation operation, we combine the raw feature information and the information in hierarchical structure. Then the fused feature map is fed to an upsampling process. In symbols, a 2D dilated convolution can be expressed in Equation (2),

(2) $$Y_{t}=\sum_{i=0}^{x}\sum_{j=0}^{y}S_{t}\left(c,x+r\times i,y+r\times j\right)$$

where parameter *r* is the dilation rate. In this process, a kernel of size k×k is enlarged to k+(k−1)(r−1) with dilated stride *r*, to obtain the output frame at the same resolution. Hence this allows us to enhance the captured context at various spatial scales.

### 3.3. Pixel Recovery

The native resolution of the frames are downsized by a certain factor, as lower spatial resolution can significantly reduce the run-time costs. We use a common empirical factor of 1/8. This downscaled resolution is conserved throughout the whole process. The feature map $Y_{t}\;\epsilon \;R^{c\times x\times y}$ is upsampled to $Y_{t}^{\prime }\;\epsilon \;R^{c\times x\times 8\times y\times 8}$. We use an upsampling kernel $w\;\epsilon \;R^{k^{2}\times 1}$ to obtain each pixel in $Y_{t}^{\prime }$.

By applying the kernel to a channel of the local feature map $\chi \;\epsilon \;R^{C\times kxk}$ centered at position *l* on $Y_{t}$, denoted by $X\;\epsilon \;R^{1\times kxk}$, the corresponding upsampled feature point $Y_{t_{l}}^{\prime }\;\epsilon \;Y_{t}^{\prime }$ of the same channel at target position *l* is obtained by $Y_{t_{l}}^{\prime }=w^{T}\times X$.

This sorts pixels from channels to the spatial dimension and without an additional layer. We replace the simple interpolation step on the network output in the GSVNET by this low-rank bilinear formulation.

## 4. Experimental Results and Analysis

### 4.1. Dataset Construction

**Figure 1:**
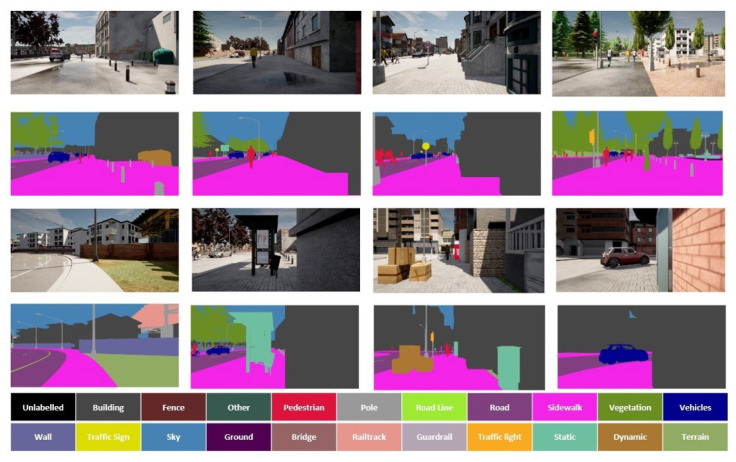
The first and third rows show some sample video frames from our agent in the CARLA urban sidewalk environment. The second and fourth rows illustrate finely annotated RGB segmentation ground truths for each corresponding frame in the upper row. The annotations are for 22 classes, as per class names and colour labels as shown in the palette. These include all the groups from the Cityscapes dataset, with an additional three classes as road lines, guard rails and static objects.

The native GSVNET model uses the Cityscapes [51] and CamVid [52] datasets for training and benchmark. The distribution of these datasets are in the context of vehicles in a road, a dissimilar collection of scenes from our target distribution. Figure 1 shows some sample snippets of synthetic scenes we develop by using the CARLA [53] simulator, with similar specifications as Cityscapes. CARLA is an open-source simulator for supporting, training, and validating autonomous driving systems in urban environments with a wide variety of sensor suits.

We generate video snippets of fixed distribution in the sidewalk preview with image capture geometries set as per our camera properties and its mounting location in our EPW. The total volume is split as 11,904/8020/10,076 for training/validation/testing. Each snippet has a resolution of 2048×1024 with 30 frames for each sequence with the 19th frame finely annotated for 22 classes. These 22 classes include all the group of classes from the Cityscapes dataset with additional crucial classes such as road lines, guard rails and static objects, (such as fire hydrants, fixed benches, fountains, and bus stops). To further enrich diversity and generalization, the scenes are captured in various visibility, weather, and intricate scenarios. Figure 1 shows some sample frames from our dataset.

### 4.2. Evaluation Metrics

For measuring the accuracy of the segmentation, pixel accuracy and variants of the Jaccard index [54] are the most popular statistic used for gauging the similarity and diversity of sample sets generated with semantic segmentation networks. The Jaccard index is also known as the mean intersection over union (mIoU) calculated as presented in Equation (3):

(3) $$\mathrm{mIoU}=\frac{1}{k}\sum_{i=1}^{k}\frac{TP_{i}}{TP_{i}+FP_{i}+FN_{i}},$$

where $TP_{i},FP_{i},FN_{i}$ are true positives, false positives, and false negatives for number of classes *k*. The predictions are accounted for frames as *l*, image pairs (k,i), where k=0 … i−1, for each frame *i* that has ground truth annotation available.

Mean pixel accuracy (mPA) is determined in Equation (4),

(4) $$mPA=\frac{1}{k}\sum_{i=1}^{k}\frac{n_{ii}}{t_{i}},$$

where $n_{ii}$ is the total number of pixels corresponding to true positives for class *i*, $t_{j}$ is the total number of pixels annotated as class *i* from *k* total number of classes. *mPA* is an intuitive metric that implies the effectiveness of individual class weights used for imbalanced class datasets to prevent overfitting of the model.

### 4.3. Implementation

We implement our network on PyTorch [55] and use the pre-trained weights SN-R18 and BN-R18 [20] from GSVNET for key-frame segmentation. At inference time, the size of the key frame is downscaled by a factor of 0.75 to facilitate the best accuracy–throughput trade-off. The segmentation output of these frames is then upsampled to the full resolution for accuracy measurement.

**Figure 2:**
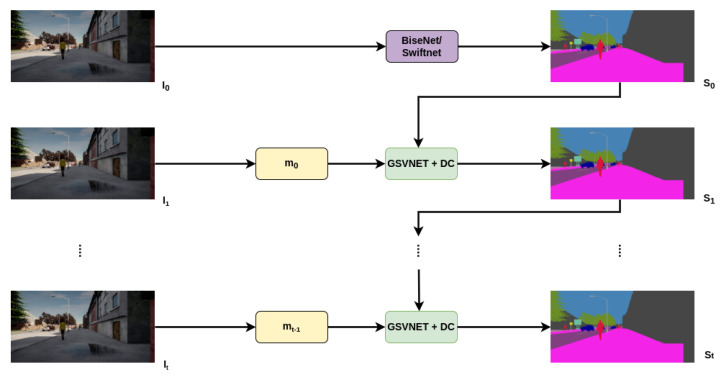
Demonstration of our proposed method. BiseNet/SwiftNet is the base segmentation network, GSVNET with dilated convolution (DC) is the propagation framework. Segmentation prediction maps S from frames I are temporally propagated until the next keyframe is reached.

Figure 2 provides an overview of our proposed method. The process begins by segmenting the first key frame to obtain a crude semantic segmentation from BiseNet/SwfitNet as a base segmentation network. Our propagation framework, a combination of GSVNET and dilated convolution processes, then temporally propagates the segmentation prediction maps from previous frames to assist in predicting the segmentation maps for the current non-key frames. At each step, the segmentation maps from GSVNET of previous frames are subject to dilated convolution to refine and enhance the segmentation maps.

The models are trained end-to-end with the training objective that involves an ordinary cross-entropy loss imposed on the final segmentation output. Similar to [20], we use stochastic gradient descent (SGD) with a momentum of 0.9 and a learning rate of 0.002, which is decreased by a factor of 0.992 every 100 iterations and set the batch size to 8 and the weight decay to 0.0005. The complete training and validation dataset are generated with CARLA.

We train our models on the MYRIA [56], with the AI–Deep Learning framework. MYRIA is composed of 366 Broadwell dual-processor compute nodes (28 cores at 2.2 GHz, 128 GB RAM) including 20 nodes, each equipped with either 4 GPU Kepler K80 (12 GB VRAM per GPU) or 2 GPU Kepler P100 (12 GB VRAM per GPU). For testing, we use NVIDIA Jetson TX1 with NVIDIA Maxwell GPU and a Quad-core ARM Cortex-A57 MPCore processor.

### 4.4. Results and Analysis

We report the accuracy measurements in terms of mIoU and mPA. All the accuracy numbers are measured at native resolution. For complexity assessment, we report the throughput in frames per second (FPS) on NVIDIA Jetson TX1 and the number of network parameters in bytes. In addition, we report the accuracy-throughput trade-off of each models.

Table 1 compares the accuracy-throughput trade-offs of GSVNET models and our models. As shown, SN-R18 from our method outperforms SN-R18 from GSVNET at the same scale factor 0.75 by 21% in mIoU, 19.4% in mPA. Likewise, our BN-R18, under similar configuration outperforms GSVNET BN-R18 by 27.1% in mIoU, 8.3%. Although there is a loss of 19.5% speed in SN-R18 and 10.9% in BN-R18, our approach still transcends in terms of mIoU with acceptable values of FPS for our application.

We also compare the complexity of our method in terms of number of network parameters between GSVNET and our method. SN-R18 from GSVNET has 47.2 M parameters for segmenting key frames and 1.6 M parameters for non-key frames, at same scale factor 0.75 and speed of 65 FPS. Our scheme, with the same configuration, at the speed of 46.5 FPS generates the same number of training parameters for key frames and non-key frames. Therefore, our method introduces no overhead and retains the key lightweight characteristic of GSVNET while improving the accuracy.

**Figure 3:**
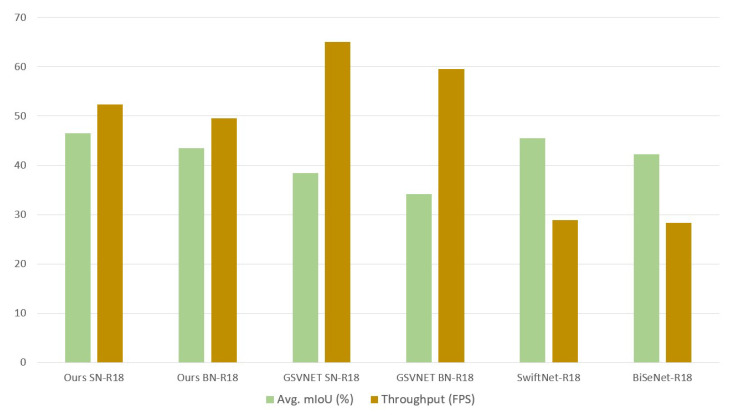
Comparison of accuracy-throughput performance using our dataset on our end hardware, NVIDIA Jetson TX1. Our models, exclusive of the pre-trained GSVNET SN-R18 or BN-R18 and SwiftNet-R18 or BiSeNet-R18, are trained and validated with our dataset.

In Figure 3, we present a fair comparison of accuracy-throughput performance. We benchmark the competing models on same hardware and configuration. Because of the lightweight temporal propagation of GSVNET, SwiftNet-R18 and BiSeNet-R18 achieve the highest through output with a considerable loss of accuracy. But in our approach, we alter this trade-off to gain in accuracy at a modest loss of speed.

**Figure 4:**
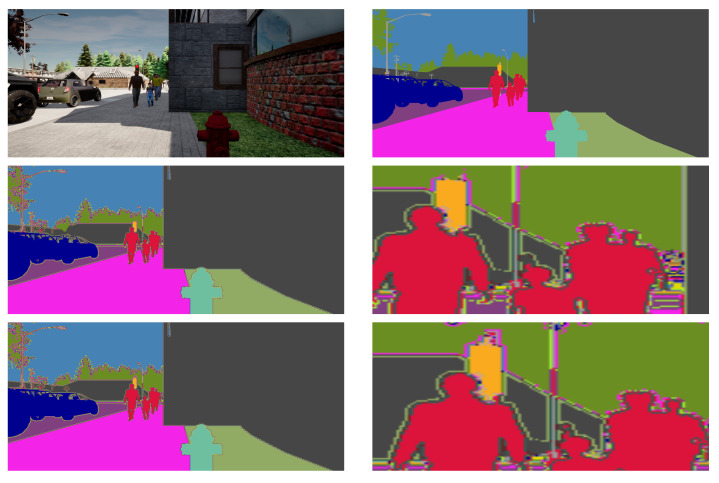
Qualitative evaluation, scenario 1. From the top down, the first row shows a sample snippet (sidewalk, traffic light, and pedestrians at far) with its ground-truth annotation. The second and third row contains the segmentation prediction maps with a zoom-in for detail depiction, from GSVNET SN-R18 and our SN-R18 models.

**Figure 5:**
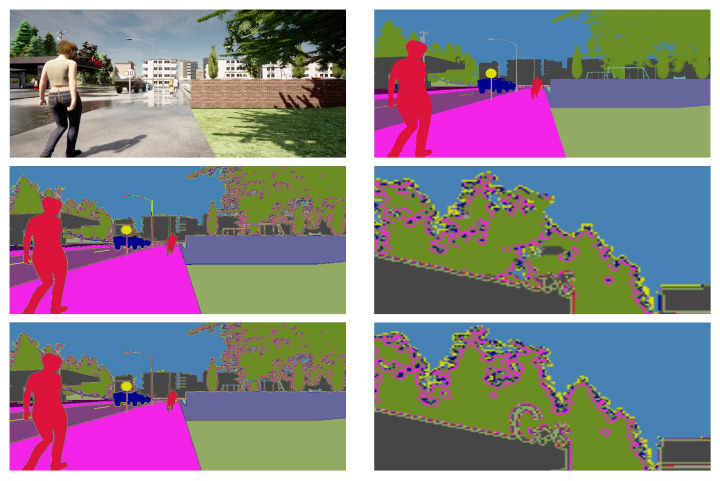
Qualitative evaluation, scenario 2. From the top down, a sample snippet (sidewalk with pedestrians, traffic signs, and contextual information of building) is presented with its ground-truth annotation, segmentation prediction maps with a zoom-in for detail depiction, from GSVNET SN-R18 and our SN-R18 models.

Figure 4 and Figure 5 shows some qualitative evaluation. The enlarged view depicts the comparison of the loss of details from the ground-truth annotation. The segmentation map from GSVNET suffers a considerable loss of information in pixel units. In Figure 4, the prediction maps from GSVNET has object boundaries distorted with a loss of information such as traffic light pole edges, pedestrians at a distance and, in Figure 5, the lost text in buildings. Because of our dilated convolution and pixel-recovery technique, our models can afford to perform better with more detailed segmentation prediction maps. As seen in the enlarged view of map from our method, the information lost with GSVNET is preserved, due to better object boundary segregation.

## 5. Conclusions and Outlook

This paper presents a light and efficient video segmentation method for visual perception in urban sidewalk environments for robotic EPWs. We propose a self-supervised approach that facilitates cross-domain applications of popular CNN models into distinct use-cases. Our approach is innovative for at least two significant reasons. First, we use an open source simulator to develop a dataset of synthetic video sequences in diverse sidewalk environments with matching target distribution to succeed data unavailability. Second, we prove the feasibility of reusing available models and possibility to alter its accuracy-through output intrinsic characteristics depending on requirements at no additional cost of computational resources. We select GSVNET, a lightweight semantic segmentation framework, and institute the necessary process without overhead to obtain better accuracy and throughput trade-off for our application.

Our initial results with GSVNET as a propagation framework and SwiftNet/BiseNet as a base segmentation model prove the feasibility of performing semantic segmentation for the new sidewalk domain. But we found this setup has an exigent accuracy-throughput characteristic that can be manipulated to improve the accuracy further by moderately taxing speed. Being a mobile robotic application, it is crucial to preserve the lightweight nature of the initial setup. Therefore, we incorporate two additional process that perform dilated convolution to refine the feature map from GSVNET with a better receptive field and a low-rank bilinear upsampling technique to obtain prediction maps with better object boundaries and detailed contextual information. With our custom dataset, our approach achieves an mIoU of 46.5 at a speed of 52.3 FPS.

In the future, we expect to expand our ablation studies towards temporal consistency (TC) of the predictions over all frames in a sequence. As TC defines measurement of consistency of object predictions subject to sudden appearance and disappearance in consecutive frames, we believe that a novel unsupervised TC metric has a strong correlation with the supervised intersection over union metrics and can help to reliably benchmark models in terms of consistency.

## Figures and Tables

**Table 1:** The accuracy and throughput comparison using our dataset with GSVNET and our method. The scale factor specifies the downscale factor for key frames in the video sequences.

Method	Model	Scale Factor	Avg. mIoU	mPA	FPS
**GSVNET**	SN-R18	0.75	38.4	65.8	65
	BN-R18	0.75	34.2	66.7	59.5
**Ours**	**SN-R18**	0.75	**46.5**	**78.6**	52.3
	**BN-R18**	0.75	**43.5**	**72.3**	49.5

## Data Availability

Not applicable.

## Abbreviations

The following abbreviations are used in this manuscript:

EPW	Electric Powered Wheelchair
ROS	Robot Operating System
ML	Machine Learning
DL	Deep Learning
RGB-D	Red, Green, Blue—Depth
ADAPT	Assistive Devices for disAbled People using robotic Technology
VSS	Video Semantic Segmentation
FSS	Fast Semantic Segmentation
SVC	Spatially Varying Convolution
SGD	Stochastic Gradient Descent
ResNet	Residual Neural Network
BiSeNet	Bilateral Segmentation Network
CNN	Conventional Neural Networks
GSVNET	Guided Spatially-Varying Convectional Network
CamVid	Cambridge-driving labeled Video dataset
SGD	Stochastic Gradient Descent
mIoU	Mean Intersection Over Union
mPA	Mean Pixel Accuracy
FPS	Frames Per Second
TC	Temporal Consistency
